# Interrogating Patterns of Cancer Disparities by Expanding the Social Determinants of Health Framework to Include Biological Pathways of Social Experiences

**DOI:** 10.3390/ijerph19042455

**Published:** 2022-02-21

**Authors:** Celina I. Valencia, Francine C. Gachupin, Yamilé Molina, Ken Batai

**Affiliations:** 1 Department of Family and Community Medicine, College of Medicine—Tucson, The University of Arizona, Tucson, AZ 85711, USA; fcgachupin@arizona.edu; 2 Division of Community Health Sciences, School of Public Health, University of Illinois at Chicago, Chicago, IL 60612, USA; ymolin2@uic.edu; 3 Department of Urology, College of Medicine—Tucson, The University of Arizona, Tucson, AZ 85724, USA; kbatai@arizona.edu

**Keywords:** cancer disparities, social determinants of health, biosocial, structural racism, BIPOC

## Abstract

The objective of this article is to call for integrating biological pathways of social experiences in the concept model of cancer disparities and social determinants of health (SDH) fields. Black, Indigenous, and People of Color (BIPOC) populations experience more negative outcomes across the cancer continuum. Social conditions are instrumental in better understanding the contemporary and historical constructs that create these patterns of disparities. There is an equally important body of evidence that points to the ways that social conditions shape biological pathways. To date, these areas of research are, for the most part, separate. This paper calls for a bridging of these two areas of research to create new directions for the field of cancer disparities. We discuss inflammation, epigenetic changes, co-morbidities, and early onset as examples of the biological consequences of social conditions that BIPOC populations experience throughout their lifespan that may contribute to disproportionate tumorigenesis and tumor progression.

## 1. Introduction

Cancer persists as one of the leading forms of disease burden in the United States (U.S.) with one in three individuals receiving a cancer diagnosis at some point in their lifetime [1]. Cancer is the second leading cause of mortality in non-Hispanic White (NHW), Black, and American Indian/Alaska Native (AI/AN) populations and is the leading cause of mortality for Latinos [2]. Excessive mortality risk has been identified in Black, Indigenous, and People of Color (BIPOC) populations for colorectal [3], cervical [4], stomach [4], liver [4], kidney [5,6], lung [7], prostate [8], and breast [9] cancers when compared with their NHW counterparts.

The cancer continuum begins with pre-diagnosis cancer prevention behaviors, including primary prevention and routine screenings, diagnosis, and survivorship, and ends with cancer mortality. Across the continuum, pronounced disparities in BIPOC communities have been identified. Examples of these patterns of difference seen in BIPOC include: less engagement in routine cancer screenings, differences in cancer treatment initiation and surgical interventions, and higher mortality rates within five years of diagnosis. A litany of potential mechanisms for these variations in cancer trends have been suggested for BIPOC populations. The relationship between social inequality and cancer disparities among BIPOC communities has been the focus of previous studies [10,11]. This body of evidence points to the social phenomenon as being instructive in better understanding unequal cancer burdens in these populations. 

To fully consider cancer disparities, there is a need to bridge the literature that focuses on social conditions that shape biological and physiological pathways to the biological pathways that increase cancer risk. This bridging highlights both the complexity and critical importance of social inequality and its effect on cancer disparities, which tandemly identifies preventable cancer risk factors, highlighting opportunities for prevention and intervention. This paper will provide an overview of the social determinants of health (SDH) framework, discuss pertinent sites of empirical evidence for BIPOC cancer disparities, and make the case that there needs to be an integrated model of biological pathways within the SDH framework for cancer disparities. From this integrated conceptualization of these bridged areas of evidence comes new horizons to move the field forward and enable new strategies for building cancer equity. 

## 2. Social Determinants of Health Framework 

We acknowledge the key roles of social and economic conditions in cancer disparities and the possibility for these conditions to be leveraged in the creation of equitable cancer outcomes. In accordance with Healthy People 2030, SDH are grouped into five domains: (1) economic stability; (2) education access and quality; (3) health care access and quality; (4) neighborhood and built environment; and (5) social and community context. These conditions encompass interrelated multi-level structures, contexts, cultures, and institutions that produce health outcomes [12,13]. SDH influences downstream factors that produce barriers and constraints on individual-level behaviors [10,12]. Examples of SDH for cancer include: not being in close proximity to low- or no-cost routine cancer screening, low health literacy leading to poor understanding of cancer care and poor quality of care [14], limited access to fruits and vegetables when residing in a food desert, not having enough food leading to smoking tobacco [15], environmental exposures such as air pollution when living adjacent to industrial areas, and absence of accessible green spaces for physical activity. Moreover, contemporary SDH are the product of larger historical legacies that shape the positionality of the individual in society via structural racism [16], redlining [17], and forced migration [18]. 

Socioeconomic position (SEP) is a notable social determinant that demonstrates the intersectionality of the domains of SDH while also being an established predictor for a wide range of negative health outcomes, including cancer disparities. The construct of socioeconomic status has multiple variations and theoretical underpinnings demonstrating its complexity. We use SEP as it encompasses the social and economic factors that shape the societal position of individuals and groups, such as income, education, and occupation, while also capturing historical origins and processes that inform societal positionality [19,20]. SEP provides a good example for exploring the interconnectedness of SDH to estimate the full magnitude of the burden of these social factors.

For cancer, SEP has been found to increase mortality risk when considered at both the individual and neighborhood level [17,21]. The intersection of SEP and race/ethnicity is key to understanding the interrelatedness of SDH in predicting health outcomes within cancer disparities. We approach race, here, as a social construct that influences the experiences and positionality of the individual. These social experiences have been found to have biological and physiological consequences [22]. The physiological consequences of race are not tied to biology but, rather, to social patterns of inequality. It is through this pathway that race/ethnicity is associated with patterns of cancer disparities. The positionality of race/ethnicity also intersects with other structural realities such as SEP, intergenerational wealth, geographies, residence, structural racism [16], and historical trauma [18]. These structural mechanisms of social inequality represent a comprehensive but not exhaustive list. Additional complexities of social inequality continue to emerge as being salient considerations for cancer disparities.

## 3. Biological Pathways of Social Experiences

**Figure 1:**
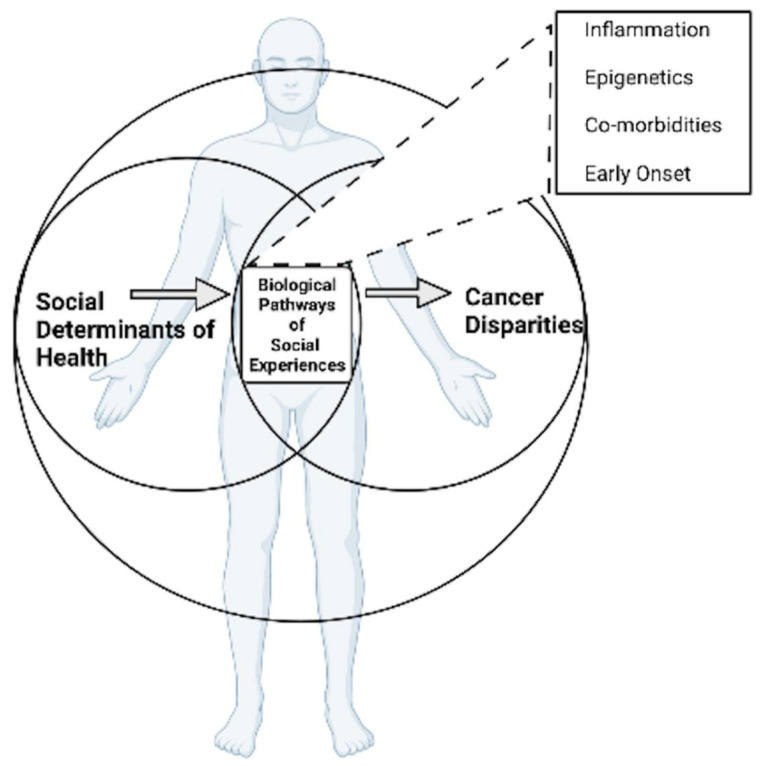
Integrated biological pathways of social experiences in concept model of cancer disparities.

Social conditions impact health in multiple capacities that range from well-being to cellular-level processes (Figure 1). Established biological pathways of social experiences include inflammation [23], telomere lengths [24], allostatic load [25,26], epigenetic changes [27] such as DNA methylation, histone modifications, and micro-RNA regulation [28,29]. The weathering hypothesis posits a theoretical framework that centers the allostatic load to be the chronic burden of social positionality across the lifespan as the underpinning of health inequality [26,30]. A study from 2021 propounded that weathering played a role in race/ethnic differences in breast cancer subtypes [25,31]. Moreover, the biological pathways of SDH should not be relegated to being solely incurred through the experiences of the individual. Biological pathways may have linkages to enduring health consequences of historical trauma, such as forced migration experienced by AI/AN [18]. We discuss inflammation, epigenetics, chronic co-morbidities, and early-onset cancers at length in this paper as these areas of research provide promising evidence for the interrelatedness of SDH, biological pathways, and cancer disparities.

## 4. Inflammation 

Inflammation has been found to be involved in every stage of the cancer continuum. Evidence suggests inflammation to be a risk factor for cancer [32], playing a role in cancer progression [33], metastases, and recurrence [34]. Increased inflammation, considered to be a proximal measure for allostatic load, often reflects psychosocial and behavioral factors. Inflammation is a key measure of experiences of stress and distress across the life course [35]. Stress, both acute and chronic burdens, has been found to lead to the downregulation of immunity and the increase in inflammation [36]. The findings on the role of stress and inflammation have been mixed; stress, however, has been found to ignite many of the cancer hallmark molecular pathways [37].

Higher levels of inflammation have been seen among BIPOC when compared with NHWs [38,39]. Higher Interleukin-6 (IL-6) levels have been found among Black adolescents [40,41]. In a study of children, there were elevated C-reactive protein (CRP) levels found among Black and Latino children when compared with White children in the National Health and Nutrition Examination Survey (NHANES), a nationally representative sample [42]. Various mechanisms for the differences in levels of inflammation have been suggested. Social context, including, but not limited to, economic, political, and environmental elements [43], provides promising insights into the disproportionate BIPOC inflammation patterns. Most inflammation studies have focused on Black populations and further research is needed to better understand rates of inflammation in other U.S. minority populations.

Socioeconomic position has been found to have various biological and physiological influences and may play a critical role in cancer disparities faced by BIPOC. Lower SEP, at the familial and individual levels, has been associated with elevated levels of inflammatory biomarkers, including CRP and other inflammatory cytokines such as IL-6 [44,45]. Additionally, childhood SEP predicted elevated CRP levels in adults [46], indicating that SEP experienced across the life course may play a role in inflammation. These biomarkers of inflammation have been found to significantly elevate the disease risks of the individual [47]. In breast cancer patients, women with a lower SEP have been found to have statistically significantly higher rates of CRP and inflammation [48]. The proinflammatory pathways associated with lower SEP are an important consideration in disentangling the role of SEP in physical health disparities [47].

Inflammation is a prominent variable for cancer mortality, with 15–20% of cancer deaths and an additional 15% of cancer deaths associated with obesity-related inflammation [49]. Inflammation coupled with excess weight and obesity increases cancer mortality risk [50]. The risk posed by inflammation is not specific to post-cancer diagnosis as elevated CRP in childhood was predictive for cancer mortality [51]. As inflammation is higher among BIPOC and those with a lower SEP, more research is needed to identify modes for intervention, especially since it is a preventable risk factor [39].

## 5. Epigenetics

The field of epigenetics provides empirical evidence demonstrating that changes to biological pathways are shaped by social experiences across the lifespan beginning in utero. The identified social experiences include, but are not limited to, stress from food insecurity, migration, psychosocial stress, and social inequality [52]. Mechanisms linking SEP and disease phenotypes are multifactorial and complex, but many studies found associations between methylations of inflammatory genes and SEP [53,54,55,56]. Maternal SEP has been found to lead to DNA methylation in placentas, which is thought to influence the health of newborns [57]. Childhood SEP has also been found to be more highly associated with DNA methylation than adult SEP [56,58]. Race/ethnicity may also play a key role in epigenetic differences across populations [59]. Low SEP has been indicated in both DNA methylation as well as in accelerated epigenetic ageing, measured with DNA methylation in the genome [60]. Emerging evidence on epigenetic age is demonstrating promising use for predicting cancer risk [61].

Cancer disparities in BIPOC associated with epigenetic changes include lung [62], breast [63,64], prostate [64], and colorectal [3]. Importantly, the cancer types associated with increased risk from epigenetic changes are also the cancer types with a heavier mortality burden in BIPOC populations. Epigenetic changes, including DNA methylation, have been posited as driving the disproportionate burden of early-onset breast cancer in Black women [63]. While the broader field of epigenetics has been considering the role of social experiences in genetic alterations, there is a gap in the epigenetic cancer literature regarding this associative relationship. There is a lack of diverse study samples in terms of both race/ethnicity and SDH to understand how social positionality may influence epigenetic modifications resulting in cancer genesis. This limits the ability to investigate the epigenetic consequences of social conditions in BIPOC cancer disparities. Future epigenetic studies in diverse study samples, undertaken in culturally respectful ways, that allow for an analysis of the intersection of race/ethnicity, SEP, and cancer outcomes are needed for prevention and intervention [65].

## 6. Complications from Co-Morbidities

Obesity is a salient consideration for cancer disparities as there are currently 13 obesity-related cancers [66]. Race/ethnicity and SEP are established independent risk factors for the development of obesity. BIPOC populations experience higher rates of obesity [67]. The underlying reason as to why race/ethnicity and SEP are independent risk factors for obesity is unclear. Some studies suggest that lower SEP individuals often reside in food environments that limit access to affordable fresh fruits and vegetables [68]. Individuals with diets high in sugar and fat are associated with obesity and increased inflammation. These types of food are often more accessible, in terms of cost and convenience, in food environments with limited produce options [67]. Food insecurity, the lack of access to adequate food [69], has been associated with the development of obesity, especially among BIPOC women [70]. In underserved areas, access to urban green spaces has been shaped by social inequality [71] creating barriers to physical activity for residents of these neighborhoods. Barriers to physical activity are notable as there is an inverse relationship between physical activity and cancer [72]. The BIPOC obesity burden then may inform trends of cancer disparities in BIPOC populations [73,74]. Obesity has not only been found to play a role in the incidence of cancers in BIPOC, but has also been found to be a factor in increased mortality rates in postmenopausal breast, colon, esophagus, and kidney cancers [67]. The biological mechanisms that may link obesity to increased risk of cancer diagnosis and mortality is uncertain but insulin resistance, altered microbiome, inflammation, and epigenetic alterations have all been suggested to play a role in this associative relationship [75,76]. More research is needed to disentangle the social elements of obesity experienced by BIPOC that may be contributing to the cancer incidence and overall negative cancer outcomes in these groups.

Diabetes is another co-morbidity that has been found to be a relevant consideration. Obesity is a risk factor in the development of both diabetes and cancer [77]. Insulin resistance, prediabetes, and gestational diabetes have all been associated with increased cancer risk, poor survivorship, and mortality [4,76]. Diabetes also has been found to be a risk factor for prostate, breast, colorectal, kidney, and liver cancers [77,78]. Each of these cancers has been found to have an excessive risk of mortality in BIPOC populations [9,79]. Cancer survivors have been found to be more likely to develop diabetes independent of established risk factors [80]. Diabetes has been found to increase mortality after cancer [77]. This makes diabetes a relevant consideration for cancer survivorship disparities as BIPOC populations are at an increased risk of Type 2 diabetes (T2D) [81,82,83]. These populations have also been found to have difficulty in T2DM disease management [84]. This makes T2D a key area of concern for BIPOC cancer disparities. 

Pre-existing and developed post-cancer diagnosis chronic obstructive pulmonary disease (COPD) has been associated with negative cancer outcomes. Chronic obstructive pulmonary disease has been identified as an independent risk factor for lung cancer [84]. Individuals that experience COPD as a cancer co-morbidity have an increased likelihood of mortality for lung, breast, renal, and colorectal cancers [85,86,87]. Patients diagnosed before the age of 39 have a substantially high risk of COPD mortality [85,88]. Risk factors for developing COPD include factors often associated with low SEP such as occupational exposures, ambient and/or household air pollution, housing conditions, and access to health care [89,90]. Use of tobacco products is the most common risk factor for COPD. The prevailing tobacco use in the literature points to an inverse relationship between socioeconomic status and tobacco use [91,92]; however, emerging evidence suggests high-income and high-education Black and Latino populations have high rates of smoking cigarettes, vaping e-cigarettes, and exposure to secondhand smoke [93]. Additionally, high-income and high-education BIPOC individuals may still be faced with issues of positionality, such as residential segregation, that places them at an increased risk of developing COPD [90]. Neighborhood-level poverty and rurality, which has also been associated with poverty [94], have both been indicated as factors that increase smoking prevalence among residents which may contribute to the development of COPD in these populations. For BIPOC populations, COPD is a notable co-morbidity in working towards equity in cancer outcomes. 

## 7. Early-Onset Cancer Disparities

There is a trend of increasing rates of early-onset cancer, defined as diagnosis before the age of 50. The observed younger median age of cancer diagnosis in BIPOC may be due young age structure in Latinos [95]; evidence suggests, however, that BIPOC individuals are experiencing a heavier burden of early-onset renal [5], lung [96], colorectal [97], and breast [98] cancers.

The research on early-onset cancers has centered on genetic predispositions that may contribute to trends among BIPOC. Batai et al. [5] argue that genomic ancestry, or ancestry related to biologic factors, might play a role in the age patterns of renal cell carcinoma in American Indian and Mexican descent populations in the U.S. Similar arguments have been made for early-onset breast cancer among Black women [99] and early-onset colorectal cancer among Black and Latino populations [100]. While we believe that genetic predisposition is an important consideration, the interaction of genes with extrinsic social factors is meaningful in understanding patterns of early-onset cancer disparities across BIPOC populations. 

Obesity has been found to be a risk factor in the early onset of gastrointestinal cancers [101] including colorectal [102,103] and breast cancer in Black women [104]. Pregnancy weight gain and birth weight have been found to increase the child’s risk of developing early-onset colorectal [105] and breast [106,107] cancers. Obese cancer patients are found to have poorer prognosis and higher rates of mortality [108,109]. Obesity-associated inflammation has been identified as the potential mechanism driving these cancer patterns. As aforementioned, BIPOC populations in the U.S. are disproportionately burdened by excess weight and obesity. Interventions for obesity prevention and reversal that consider social factors are a potential strategy as obesity may be a major driving factor.

## 8. Conclusions

### New Directions for the Field of Cancer Disparities

The available empirical evidence demonstrates the linkages of the formative role of social experiences across the lifespan on biological pathways [110]. Historical trauma and social exposures during childhood and adolescence are notable social experiences in BIPOC populations that need to be further evaluated in the field of cancer disparities. Biological pathways that may be shaped by historical trauma and social exposures may place BIPOC at an increased risk of cancer disparities, but currently there is a lack of evidence to fully realize the extent of this impact. Research on biological pathways has primarily focused on Black populations who suffer from unique and severe forms of oppression. Limited empirical evidence of biological pathways of social experiences has been considered in other populations of color. Diverse study samples that disaggregate people of color and immigrant communities, groups who have been historically captured monolithically, is needed to understand the biological impacts of social experiences in these groups. These pathways should include an investigation of the patterns of immunological and inflammatory dysfunction that have been found to increase the risk of developing cancers such as Hemoglobin A1c [111]. The field of epigenetics provides another promising new opportunity to disentangle the impact of contemporary and historical social inequality experienced by BIPOC. 

To curb cancer disparities with this integrated SDH approach, there must be full consultation and mutually beneficial partnership with BIPOC communities. As social institutions may persist in excluding historically marginalized communities, the extent of the biological and physiological implications can only be fully understood through productive conversation, with a vision of shared governance and open science, that encourages transparency between the researcher and communities. This centers participants in ways that create a possibility for participants to be part of the decision-making process. These strategies include, but are not limited to: consultation with tribal governments; acknowledgment of data sovereignty; and research dissemination in the form of community meetings, town halls, and similar meetings [112]. Training should provide information on community science techniques to provide a foundation for true collaborative partnerships as well as approaches for involvement with local policy makers to shape policies in response to the evidence created through these research partnerships. 

Expanding the scope of the SDH framework to include an integrative consideration of the biological pathways of social experiences contributes to positive expansion in the field of cancer disparities. The integrated framework lends itself to a greater understanding of the magnitude of an individual’s positionality across the life course, the embodiment of social inequality in the form of epigenetic changes and physiological dysfunction, and areas of social inequality that occur disproportionately in BIPOC communities. Moreover, this framework provides insights into the interplay between each of these factors that translates into cancer disparities in BIPOC populations. Approaching these issues in partnership with BIPOC communities provides the scientific evidence that can be utilized to work towards structural changes in their context. From this, community members have the opportunity to advocate for scientifically informed policies that align with their lived realities to target contexts of building health equity by tackling SDH that disproportionately influence disparities.

## Data Availability

Not applicable.
